# TMEA, a Polyphenol in *Sanguisorba officinalis*, Promotes Thrombocytopoiesis by Upregulating PI3K/Akt Signaling

**DOI:** 10.3389/fcell.2021.708331

**Published:** 2021-08-17

**Authors:** Hong Li, Xueqin Jiang, Xin Shen, Yueshan Sun, Nan Jiang, Jing Zeng, Jing Lin, Liang Yue, Jia Lai, Yan Li, Anguo Wu, Long Wang, Dalian Qin, Feihong Huang, Qibing Mei, Jing Yang, Jianming Wu

**Affiliations:** ^1^School of Pharmacy, Southwest Medical University, Luzhou, China; ^2^State Key Laboratory of Biotherapy and Cancer Center, West China Medical School, Sichuan University, Chengdu, China; ^3^School of Pharmacy, Chengdu University of Traditional Chinese Medicine, Chengdu, China; ^4^Medical Research Center, The Third People’s Hospital of Chengdu, Chengdu, China; ^5^The Key Laboratory of Medical Electrophysiology, Medical Key Laboratory for Drug Discovery and Druggability Evaluation of Sichuan Province, Luzhou Key Laboratory of Activity Screening and Druggability Evaluation for Chinese Materia Medica, Ministry of Education of China, Institute of Cardiovascular Research, Luzhou, China

**Keywords:** thrombocytopenia, *Sanguisorba officinalis* L., PI3K, megakaryocyte differentiation, 3, 3′, 4′-trimethylellagic acid

## Abstract

Thrombocytopenia is closely linked with hemorrhagic diseases, for which induction of thrombopoiesis shows promise as an effective treatment. Polyphenols widely exist in plants and manifest antioxidation and antitumour activities. In this study, we investigated the thrombopoietic effect and mechanism of 3,3′,4′-trimethylellagic acid (TMEA, a polyphenol in *Sanguisorba officinalis* L.) using *in silico* prediction and experimental validation. A KEGG analysis indicated that PI3K/Akt signalling functioned as a crucial pathway. Furthermore, the virtual molecular docking results showed high-affinity binding (a docking score of 6.65) between TMEA and mTOR, suggesting that TMEA might target the mTOR protein to modulate signalling activity. After isolation of TMEA, *in vitro* and *in vivo* validation revealed that this compound could promote megakaryocyte differentiation/maturation and platelet formation. In addition, it enhanced the phosphorylation of PI3K, Akt, mTOR, and P70S6K and increased the expression of GATA-1 and NF-E2, which confirmed the mechanism prediction. In conclusion, our findings are the first to demonstrate that TMEA may provide a novel therapeutic strategy that relies on the PI3K/Akt/mTOR pathway to facilitate megakaryocyte differentiation and platelet production.

## Introduction

Thrombocytopenia is a common hematological disease characterized by a peripheral blood platelet count of less than 100 × 10^9^/L. In the clinic, this disease is divided into primary and secondary subtypes (Ghoshal and Bhattacharyya, 2014; Eto and Kunishima, 2016; Mones and Soff, 2019). Primary thrombocytopenia is usually caused by conditions such as adverse drug reactions, alcohol abuse, surgery, infection, hepatopathy, nephropathy, and pregnancy, which lead to excessive depletion of peripheral blood platelets and megakaryocyte maturation disorder (Stentoft, 2016; Gudbrandsdottir et al., 2020). Secondary thrombocytopenia, also known as acquired thrombocytopenia, follows multifold complex diseases such as chronic lymphocytic leukemia, rheumatoid arthritis, and systemic lupus erythematosus (Mojadidi et al., 2016; Greenberg, 2017; Castro, 2017). Currently, the main therapies for thrombocytopenia comprise drug medications, platelet transfusion and improved diet quality in the clinic (Liebman and Pullarkat, 2011; Lakshmanan and Cuker, 2012). However, platelet transfusion has been associated with allergic reactions and febrile nonhemolytic reactions (Kaufman et al., 2015), while an improved diet might merely as an adjunct to treatment. Drug regimens are still the main therapy for thrombocytopenia. Unfortunately, the agents used to treat thrombocytopenia, such as cytokines, glucocorticoids, and immunoglobulin, have severe adverse reactions, do not prevent high recurrence rates, and show low treatment tolerance (Deshayes and Godeau, 2020). Therefore, it is necessary to explore a novel approach to platelet recovery with high efficacy and low toxicity.

Currently, active compound identification and isolation from traditional Chinese medicinal plants are playing vital roles in novel drug development. As secondary metabolites in raw plant materials, polyphenols are structurally typified by benzene derivatives with multiple hydroxyl substitutions and mainly comprise flavonoids, lignans, tannins, astragalosides, ellagic acid homolog, gallic acid homologs, and curcumin (Croft, 2016; Rothwell et al., 2017). These substances are capable of reducing oxidative stress, suppressing tumour progression, and preventing cardiovascular events, which may indicate their druggability (Scalbert et al., 2005; Poklar Ulrih, 2017; Tresserra-Rimbau et al., 2018; Liang et al., 2020).

Dried root of *Sanguisorba officinalis* L. (SO), a common herbal material, has hemostatic and astringent efficacies for treating hemorrhagic diseases in the clinic. Increasing pharmacological evidence has shown that an SO regimen can promote megakaryocyte maturation and be beneficial for thrombocytopenia remission (Gao et al., 2014). However, its key component with platelet-elevating activity remains unidentified, impeding further understanding of the mechanism of SO action. However, polyphenols were discovered to moderate irradiation-induced megakaryocyte apoptosis (Xu et al., 2018), suggesting a potential ability of polyphenols to induce thrombopoiesis, which coincides with our preliminary experimental results. Here, 3,3′,4′-trimethylellagic acid (TMEA), a representative SO polyphenol, may exert a therapeutic impact on thrombocytopenia according to the existing evidence. Nevertheless, it is imperative to confirm the prothrombopoietic effect of TMEA and clarify its molecular mechanism.

Network pharmacology offers a feasible approach for systematically investigating the complex relationship between drugs and diseases through systems biology and network analysis methods (Casas et al., 2019; Luo et al., 2020). According to the disease-gene-drug correlation, network pharmacology can be applied to explore potential therapeutic targets for drugs, generally improving the efficiency of drug discovery (Hao and Xiao, 2014; Jiang et al., 2019). In addition, molecular docking simulation is an emerging strategy in computer-aided drug design based on receptor characteristics and receptor and drug molecules interactions (Saikia and Bordoloi, 2019; Santos et al., 2019; Pinzi and Rastelli, 2019; Kaur et al., 2019; Aucar and Cavasotto, 2020). The binding abilities are scored in multiple ways. The hub proteins and signalling pathways can then be deduced by visualizing small molecules interacting with target proteins. In summary, the use of these two *in silico* techniques may provide reliable insight into and understanding of the mechanism of compound action because of the available sources with large amounts of data.

In the present study, we probed the molecular mechanism of TMEA action on thrombocytopenia via a network pharmacology approach, which might underpin its potential therapeutic effects. The binding of TMEA with the hub protein mTOR was verified by molecular docking simulation. *In vitro* and *in vivo* investigations on TMEA activity were designed to verify these predicted results. Our findings showed that TMEA could activate the PI3K/Akt/mTOR signalling pathway to promote platelet production. In summary, this study presents helpful ideas by reporting a combined computational analysis with an experimental trial to explore the thrombopoiesis-promoting effect and underlying mechanism of TMEA in the potential treatment of thrombocytopenia.

## Materials and Methods

### Polyphenol TMEA Preparation

As previously reported (Bai et al., 2019), TMEA was prepared by silica gel column chromatography. In brief, 70% ethanol SO extract was partitioned using methylene chloride (CH_2_Cl_2_). The CH_2_Cl_2_ fraction was subjected to chromatographic isolation by silica gel and eluted with such solution systems in different proportions as petroleum ether (PE)-acetic ether (EAC) (8:2), PE-EAC (6:4), PE-EAC (8:2), and PE-EAC (10:0), successively. TMEA was obtained in PE–EAC (8:2) eluted solution. An UPLC (Exion) – QTOF (X500R) – MS system (SCIEX, Massachusetts, United States) was employed for the analysis of TMEA. The sample injection volume was 5 μL. Phenomenex Kinetex C_18_ column (100 mm × 2.1 mm, 2.6 μm, 100 Å) was used for the chromatographic separation at column temperature of 40°C. Mobile phase A [0.1% formic acid-water (V/V) mixture] and mobile phase B [0.1% formic acid-acetonitrile (V/V) mixture] were delivered at a flow rate of 0.3 mL/min for gradient elution as follows: 5% B from 0 to 2.00 min, 5% to 70% B from 2.01 to 18.00 min, and 70% to 100% A from 18.01 to 20.00 min. Ultraviolet (UV)-visible (vis) detection was performed at a wavelength of 247 nm. The mass-spectrum assay was optimized in negative ion mode. The ion spray voltage was set at -4.5 kV, and the interface temperature was set at 550°C. Data were obtained in scan mode from m/z 60 to 2000 Da and further analysed using SCIEX OS B.1.4.

### Drug-Likeness Prediction

Lipinski’s rule-of-five is used for screening potential oral drugs in humans on the basis of a drug-likeness assessment. To explore the drug-like properties of TMEA, we uploaded the SMILES format for TMEA [COC1 = C(OC)C2 = C3C(= C1)C(= O)OC1 = C(OC)C(O) = CC(C(= O)O2) = C31] into the SwissADME server,^1^ a web-based tool used for evaluating the pharmacokinetics, drug-likeness, and medicinal chemistry friendliness of small molecules (Daina et al., 2017). Screening was performed with the default parameters.

### Target Prediction

We retrieved TMEA-matching structural information from the PubChem database^2^ and then input the structural formula into the SwissTargetPrediction database^3^ to obtained the compound-related targets (Gfeller et al., 2014; Hähnke et al., 2018). Other alternative targets of TMEA action were obtained from the PharmMapper platform^4^ on the basis of a three-dimensional (3D) TMEA structure (Forouzesh et al., 2019). The gene targets linked to TMEA activity were thereby identified as ‘‘drug targets’’ through target data integration without repetition and nonhuman sources. Thrombocytopenia-relevant targets were obtained as ‘‘disease targets’’ from the GeneCards^5^ and DisGeNET^6^ databases after duplicate removal (Rebhan et al., 1998; Piñero et al., 2017). Ultimately, the mapping of “drug targets” onto “disease targets” led to the identification of the shared targets engaging in compound-target-disease interrelations.

### Protein-Protein Interaction (PPI) Network Construction and Hub Target Screening

To appraise the interactions of the targets in a network, we uploaded the information on the shared targets described above to the STRING_v11.0 database^7^ with the species defined as “Homo sapiens” (Szklarczyk et al., 2017). In a STRING network analysis, a confidence score of greater than 0.4 indicates credible interrelations among proteins. Accordingly, the eligible PPI data were input into Cytoscape_v3.7.1 software for visibility optimization and topology analysis of the PPI network. The following criteria for determining interconnective nodes assisted in identifying the hub targets (nodes) of TMEA action in thrombocytopenia: degree values greater than the corresponding medians, betweenness centrality and closeness centrality values greater than twice the corresponding medians.

### Gene Ontology Term and Kyoto Encyclopedia of Genes and Genomes Pathway Enrichment Analyses

Gene Ontology (GO) term and Kyoto Encyclopedia of Genes and Genomes (KEGG) pathway enrichment analyses were performed to decipher the functions of differentially expressed genes, which were also used to predict the potential mechanism of drug action. We entered the gene symbols of the common targets into the Database for Annotation, Visualization and Integrated Discovery (DAVID)_v6.8, a database with integrated GO and KEGG modules (Huang da et al., 2009). The GO analysis reflected a three-category evaluation of TMEA action, including the TMEA influenced genes in the biological process (BP), molecular function (MF), and cellular component (CC) categories. The KEGG analysis was performed for target-protein-related pathway screening to discover the key mechanism underlying TMEA-driven recovery from thrombocytopenia. The results from the pathway enrichment analyses were prepared for visualization as a bubble diagram using the OmicShare platform.^8^

### Molecular Docking Simulation

The use of virtual molecular docking can help comprehend the interaction process between a small-molecule compound and its target protein via *in silico* visual simulation. Thus, to observe the direct binding of TMEA with the hub targets identified in the PPI network, we performed the following procedure: First, we derived the 3D TMEA structure and crystal structures of the hub proteins [namely, TLR2 (PDB ID: 1FYW) (Ve et al., 2017), SRC (PDB ID: 1YOJ) (Breitenlechner et al., 2005), mTOR (PDB ID: 4JT6) (Yang et al., 2013), and ABL1 (PDB ID: 5NP2) (Merő et al., 2019)] from the PubChem and RCSB Protein Data Bank^9^ databases. Second, we removed any co-crystallized ligands from these 4 proteins, and water molecules were removed from the protein structures after assessment of the X-ray diffraction structures. Third, hydrogen atoms were added to the protein structures, in which the amino acid side chains were fixed. Finally, for virtual TMEA-protein interaction imaging, Surflex-Dock (SFXC) was adopted as the docking mode after protein structure preparation. Surflex-Dock scores (total scores) denote binding affinities.

### Molecular Dynamics (MD) Simulation

To dynamically observe the efficient flexible docking of each small molecule to its target proteins, we implemented MD simulations using AMBER_18. In preparation for this experiment, the molecular mechanics method was adopted to optimize the protein complex system. The general AMBER force field (GAFF) was used for the ligands, and the ff14SB force field was employed for the protein (Vaijanathappa et al., 2019). All protein-ligand complex systems were immersed in a box (with a 12.0-Å boundary) in the TIP3P water model and then neutralized by the addition of Na+ or Cl- counter ions.

The MD simulations were carried out using the PMEMD.mpi and PMEMD.cuda modules in the AMBER_18 package. Initially, several minimization steps were conducted with these systems in place to prevent possible steric crashes. Then, each system was gradually heated from 0 to 300 K during the heating stage and maintained at 300 K during the subsequent equilibrium and production stages. A time step of 2 fs was used for the heating stage, the equilibrium stage, and the entire production stage. A periodic boundary condition was employed to maintain constant temperature and pressure ensembles. The pressure was set to 1 atm and controlled by the anisotropic (*x*-, *y*-, *z*-) pressure scaling protocol with a pressure relaxation time of 1 ps. The temperature was regulated using Langevin dynamics with a collision frequency of 2.0 ps^–1^. The particle mesh Ewald (PME) method was adopted to handle long-range electrostatics, and a 10-Å cut-off was set to render real-space interactions. All covalent bonds involving hydrogen atoms were constrained with the SHAKE algorithm. Each system was subjected to a 25-ns MD simulation, and the trajectory of the simulated systems was saved every 100 ps.

For the saved trajectories of MD simulations, the MM/GBSA method was used to calculate the binding energies of the receptors exposed to each ligand. A total of 100 snapshots were extracted from 20 to 25 ns to calculate the mean binding energy. The formula is as follows:

$$\Delta \,E_{\mathrm{bind}}=\Delta \,E_{\mathrm{MM}}+\Delta \,E_{\mathrm{SOL}}=\Delta \,E_{\mathrm{MM}}+\Delta \,E_{\mathrm{GB}}+\Delta \,E_{\mathrm{SA}}$$

Where Δ*E*_bind_ is the binding energy and Δ*E*_MM_ denotes the sum of the molecular mechanical energies in a vacuum, which can be further categorized into contributions from electrostatic, van der Waals, and internal energies. This term was computed using the molecular mechanics method. Δ*E*_SOL_ is the solvation energy, which includes the polar solvation energy (Δ*E*_GB_) calculated with the generalized born (GB) approximation model and the nonpolar part (Δ*E*_SA_) obtained by fitting the solvent accessible surface area (SASA) with linear combinations of pairwise overlaps (LCPOs). Additionally, the energies of each residue were decomposed into the backbone and side-chain atoms. The energy decomposition can be analysed to determine the contributions of the key residues to binding.

### Cell Culture

HEL cells were purchased from American Type Culture Collection (Manassas, VA, United States). The cells were cultured in complete RPMI 1640 medium (Gibco, Thermo Fisher Scientific, Waltham, MA, States) containing 10% foetal bovine serum (FBS, Gibco, Thermo Fisher Scientific, Waltham, MA, States), 100 U/mL penicillin, and 100 μg/mL streptomycin (Beyotime, Sichuan, China) at 37°C in a humidified incubator with a 5% CO_2_ atmosphere.

### Megakaryocyte-Like Cells Count

HEL cells were seeded into 6-well flat-bottomed plates at an initial cell density of 4 × 10^4^ cells/well. In addition to being sustained in the complete medium and under the same incubator conditions described above, the cells were harshly treated with 10 μM and 20 μM TMEA to induce their differentiation into megakaryocytes. The medium was replaced every two days, and the TMEA containing medium was replaced to the TMEA group. Cell size increasing is one of the markers of megakaryocyte maturation (Limb et al., 2015; Huang et al., 2016), thus we counted cells that were more than twice larger than undifferentiated HEL cells. The megakaryocyte-like cells whose diameters were twice larger than undifferentiated HEL cells were counted under a microscope at 100× magnification on the 8th and 12th days of treatment. Three fields were randomly selected for mature cell counting and as the basis for statistical analyses.

### Giemsa Staining

Giemsa stain was applied to observe the cell morphology under a microscope (Stockert et al., 2014). For that purpose, HEL cells were inoculated in 6-well plates and incubated overnight in fresh complete medium. The cells were treated with 10 μM and 20 μM TMEA in separate batches. After incubation for 8 and 12 days, the cells were washed with PBS and fixed with 100% precooled methanol. Freshly prepared 5% Giemsa solution (Solarbio, Beijing, China, 2018126) was added to each well, and then, the cells were stained at room temperature for 15 min. Multiploid cells were captured at 400× magnification under a microscope.

### Phalloidin Staining

HEL cells were immobilized at room temperature with 4% paraformaldehyde solution (Biosharp, Anhui, China, 1810473) in phosphate-buffered saline (PBS) on the 8th and 12th days of treatment. The cells were rinsed with PBS and permeated with 0.5% Triton X-100 solution. After washing again with PBS, TRITC-labelled phalloidin working solution was allowed to infiltrate into the cells on glass slides. The cells were incubated in the dark at room temperature and then washed again with PBS. The nuclei were restained with DAPI solution (100 nM). The images were observed under a fluorescence microscope.

### DAPI Staining for High-Content Cell Imaging

After 12 successive days of drug intervention, the HEL cells were centrifuged and prepared for subsequent staining after the supernatant was removed. DAPI staining solution (Beyotime, Sichuan, China, 1207181412) and a 4% paraformaldehyde fixative solution (Biosharp, Anhui, China, 1810473) were mixed at a volumetric ratio of 1:100 for dyeing cell nuclei. The cells were incubated in the dark with the dye solution for 15 min and then analysed with a high-content cell imaging analysis system (Molecular Devices, ImageXpress Micro 4).

### Flow Cytometry Analysis of CD41 and CD42b Expressions

HEL cells were seeded into 6-well flat-bottomed plates at an initial cell density of 4 × 10^4^ cells/well. The cells were treated with 10 μM and 20 μM TMEA for 4,8, and 12 days. The medium was added every two days, and the TMEA containing medium was added to the TMEA group. The cells harvested at different time points were washed once with ice-cold PBS and subsequently incubated with 3 μL of CD41-FITC (BioLegend, United States, FHF0411) and 3 μL CD42b-PE (BD, United States, 555473) in 100 μL PBS in the dark for 30 min. Then the cells were re-suspended in 400 μL of PBS, and submitted to the analysis by a FACSVerse flow cytometer (BD Biosciences, SanJose, CA, United States). CD41 expression was assessed using the fluorescein isothiocyanate (FITC) channel, whileCD42b level was examined using the PE channel. The forward and side scatters were used to eliminate the disturbance from cellular fragments. The quantitation of the test biomarker expressions was based on the percentage of CD41^+^/CD42b^+^ cells.

### Assay for Reactive Oxygen Species (ROS) Level

HEL cells were seeded into 6-well flat-bottomed plates at an initial cell density of 4 × 10^4^ cells/well. The cells were treated with 10 μM and 20 μM TMEA for 4,8, and 12 days. The cells harvested at different time points were centrifuged at 1500 r/min for 3 min, re-suspended in 0.5 mL of serum-free medium containing 10 μM DCFH-DA (Beyotime, Sichuan, China, S0033), incubated in the dark for 20 min under a gentle mixing at 5 min intervals. The cells were subsequently washed with serum-free medium twice, resuspended in 500 μL of PBS, and submitted to the analysis of intracellular oxidative species (ROS) using a FACSVerse flow cytometer (BD Biosciences, San Jose, CA, United States) under FITC channel. The quantitation of the test DCF was based on the corresponding mean fluorescence.

### Cell Apoptosis Rate Analysis

Cell apoptosis rate was measured by flow cytometry using the Annexin V-FITC/PI Apoptosis Detection Kit (Vazyme, Nanjing, China, A211). Briefly, HEL cells were seeded in 6-well plates at a density of 4 × 10^4^cells/well. The cells were treated with 10 μM and 20 μM TMEA for 4, 8, and 12 days. After treatment, cells were harvested for apoptosis detection according to the manufacturer’s instructions. Flow cytometric analysis was then carried out by using a FACSVerse flow cytometer (BD Biosciences, San Jose, CA, United States). Data acquisition and analysis were performed by using the Flowjo software (BD Biosciences, San Jose, CA, United States).

### Immunofluorescence Assay

HEL cells were collected after drug intervention and fixed with 4% paraformaldehyde fixative solution at room temperature. Subsequently, we washed the cells three times with PBS, added 0.5% Triton X-100 to perforate the cell membranes within 10 min, and blocked this process by adding 5% BSA at room temperature. Then, anti-GATA-1 and anti-NF-E2 antibodies were incubated with the cells overnight at 4°C. After washing with PBS, the cells were co-incubated with DAPI to observe the nuclear translocation of the transcription factors GATA-1 and NF-E2 *via* fluorescence confocal microscopy.

### Western Blot

On the 4th, 8th, and 12th days of TMEA treatment, the HEL cells were harvested and lysed with 1× RIPA lysis buffer (Cell Signaling Technology, Beverly, MA, United States) containing EDTA-free protease inhibitor cocktail (TargetMol, Shanghai, China). Total proteins were extracted from the cells. After heating at 95°C for 10 min, the protein samples were electrophoresed and transferred to PVDF membranes (Millipore, Darmstadt, Germany). Then, the membranes were rinsed twice in PBS-Tween 20 (PBST) and immersed in 5% skim milk powder solution in PBST with gentle shaking. After 2 h of blocking, the membranes were washed in PBST 3 times and incubated overnight at 4°C separately with the indicated primary antibodies: anti-PI3K, anti-phosphorylated-(p-)PI3K, anti-Akt, anti-p-Akt, anti-mTOR, anti-p-mTOR, anti-P70S6K, anti-p-P70S6K, anti-GATA-1, or anti-NF-E2, and anti-β-actin (Cell Signaling Technology, Danvers, MA, United States). Subsequently, these membranes were washed 3 times and incubated with horseradish peroxidase-labelled secondary antibody. After 1 h of sealing, protein bands in the membranes were visualized using UltraSignal^TM^ ECL western blotting detection reagents (4A Biotech Co., Ltd, Beijing, China) and a ChemiDoc MP Imaging System (Bio-Rad, Hercules, CA, United States). These bands were quantitatively analysed by ImageJ software (National Institutes of Health, Bethesda, MD, United States). The relative image intensities of the target proteins to β-actin positively manifested their expression.

### Animals and X-Ray Modeling

Specific pathogen-free (SPF) Kunming mice, evenly composed of one-half male and one-half female mice weighing 20.0 ± 2.3 g, were purchased from Dashuo Experimental Animal Research Center (Chengdu, China). These mice were bred in specific-pathogen-free laminar flow cabinets under a 12-h light/dark cycle. The mice were fed standard diets and allowed to drink freely. The ambient temperature was controlled at (24 ± 2) °C. All experimental processes were approved by the Ethics Committee of Southwest Medical University (Licence No. 20170341). After acclimation for 7 days, the animals were randomly assigned to 4 groups: the control group, X-ray (model) group, X-ray + TPO (3000 U/kg, positive control) group, and X-ray + TMEA (10 mg/kg, treatment) group. Except for those in the control groups, the mice were X-ray-irradiated at a single 4-Gy dose to induce experimental haematopoietic dysfunction. Notably, the mice in the X-ray-exposed groups were tested to ensure relative uniformity of baseline levels of peripheral blood cells before drug administration.

### Peripheral Blood Platelet Assay

After irradiation-induced modelling, the animals in the control and model groups were intraperitoneally injected with normal saline. The mice in the positive control group were administered recombinant human TPO injection intraperitoneally. TMEA was intraperitoneally injected into the mice in the treatment group for therapeutic evaluation. All these injections were performed once per day. Subsequently, we collected 40-μL whole blood samples from the retro-orbital venous plexus of each mouse initially and on the 4th, 7th, 10th, 14th, and 17th day, and these samples were mixed and diluted by a 160-μL diluent for blood cell analysis. Complete blood cell counts were determined using an automatic haematology analyser (Sysmex XT-2000iV, Kobe, Japan).

### Statistical Analysis

The data acquired in this study were statistically analysed using GraphPad Prism 8.0 (GraphPad Software Inc., La Jolla, CA, United States). The data are expressed as the means ± standard deviation ($\bar{x}\pm S\,D$). Statistical significance was assessed using two-tailed Student’s *t*-tests or one-way analysis of variance (ANOVA). In all cases, a *P*-value < 0.05 was considered to indicate a significant difference.

## Results

### Identification of TMEA

**FIGURE 1:**
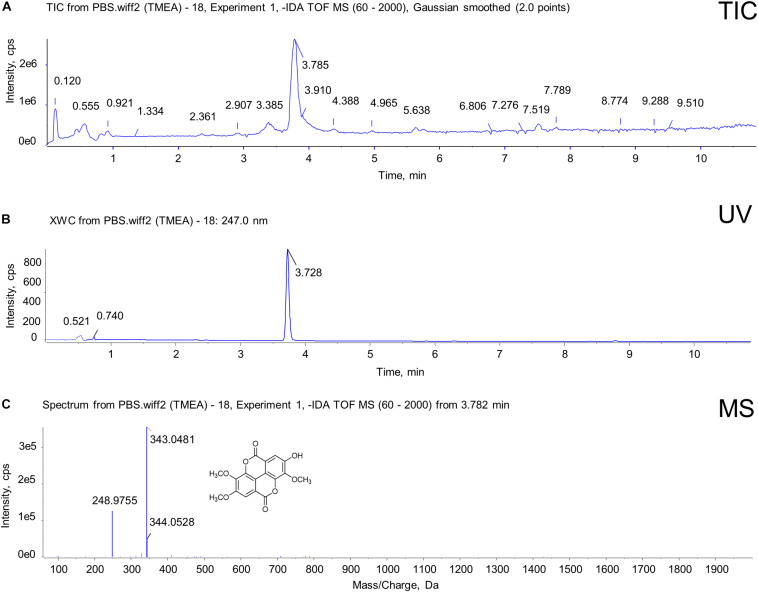
Identification of TMEA from SO roots using UPLC-QTOF-MS. **(A)** Total ion chromatogram (TIC) with 247-nm UV for TMEA. **(B)** UV chromatogram at 247 nm for TMEA. The retention time of TMEA was 3.782 min. **(C)** Diode array detection (DAD)-coupled chromatogram with 247-nm UV for TMEA. The relative molecular mass was 343.0481 [M-H]–. The structure of TMEA is shown in this figure panel **(C)**.

UPLC-QTOF-MS was used to confirm the molecular information on TMEA obtained from analyses of the SO extract. As shown in Figure 1, the retention time of TMEA was 3.785 min, and its relative molecular mass was 343.0481 [M-H]–, which partially reflects the structural characteristics of the TMEA molecule. This result suggests that TMEA may undertake SO activity in part, serving as an active component in this herbal material.

### Molecular Properties of TMEA

Drug-likeness assessment is vital for predicting latent therapeutic compounds. From Lipinski’s rule of five (Ro5), a druggable compound likely meets the following requirements: molecular weight from 180 to 500 Daltons, polar surface area (PSA) less than or equal to 140 Å^2^, a n-octanol/water partition coefficient (XLogP3) less than 5, and the number of hydrogen-bond donors and acceptors less than 5 and 10, respectively (Chagas et al., 2018). Our results showed that the properties of the TMEA molecule were in line with those of the Ro5. It may be considered a lead compound for structurally optimized drug development (Table 1).

**TABLE 1:** Molecular properties of TMEA.

**Property**	**Value**
Canonical SMILES	COC1C(OC)CC2C3C1OC(= O) C1C3C(OC2 = O)C(C(C1)O)OC
Formula	C_17_H_12_O_8_
Molecular weight (g/mol)	344.27
Rotatable bonds	3
H-bond acceptors	8
H-bond donors	1
TPSA (A^2^)	108.34
XLOGP3	2.08
Bioavailability Score	0.55

### Identification of Potential Therapeutic Targets

**FIGURE 2:**
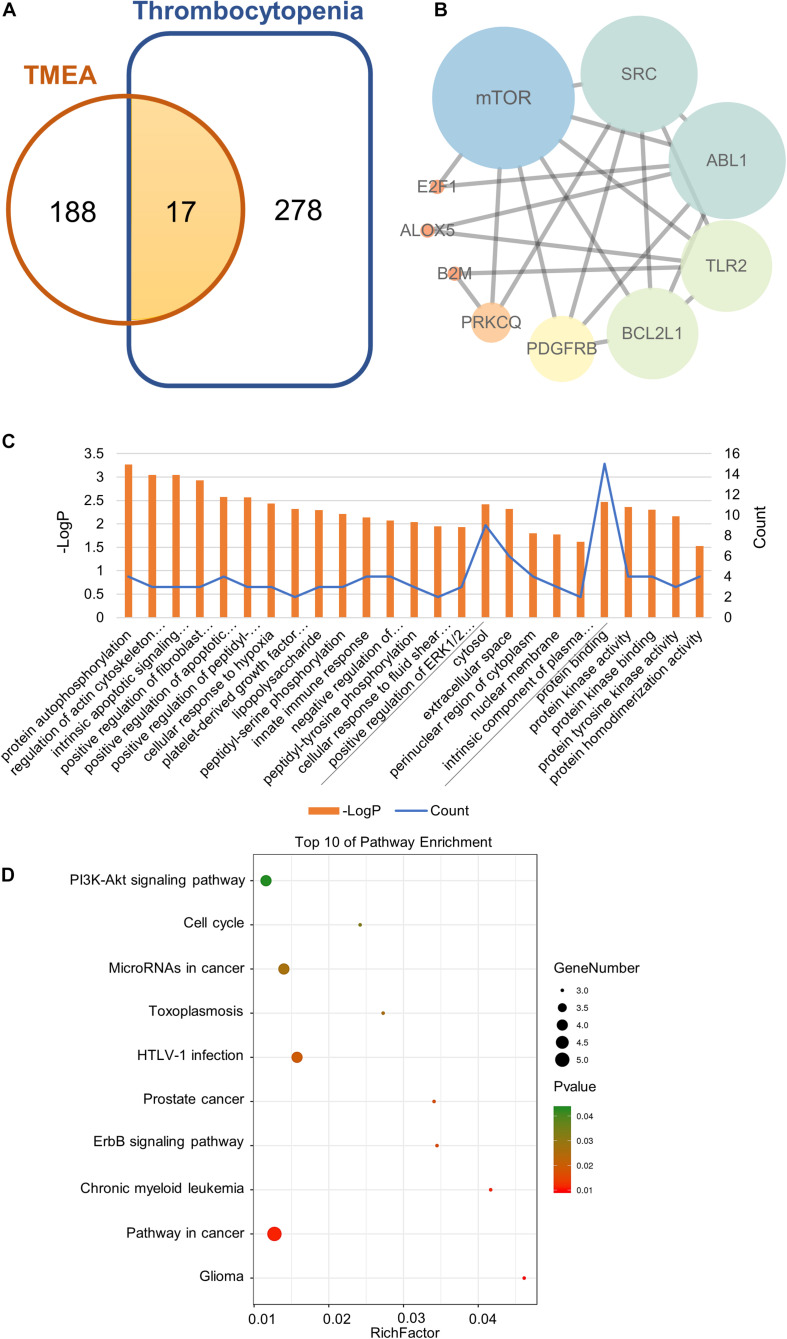
Network pharmacology analysis of TMEA activity in thrombocytopenia treatment. **(A)** Venn diagram of the shared targets of TMEA and in thrombocytopenia. **(B)** Protein-interactive (PPI) network based on the targets for TMEA in the treatment of thrombocytopenia. **(C)** GO enrichment analysis of the hub targets for thrombocytopenia treatment with TMEA. The 15 BP, 5 CC, and 5 MF terms with the greatest enrichment are listed in ascending order of P-values. **(D)** The 10 KEGG pathways enriched with hub targets for TMEA in treating thrombocytopenia. The bubble size indicates the number of enriched genes; its colour signifies the corresponding P-value. The rich factor denotes the ratio of the target genes in a pathway to all the annotated genes in this pathway. The colour of the bubble is related to the P-value.

To acquire the “drug targets” and the “disease targets,” we separately integrated the TMEA-related targets obtained from SwissTargetPrediction with PharmMapper analyses and the thrombocytopenia-associated targets obtained from GeneCards and DisGeNET. The results revealed that 105 targets were found in the SwissTargetPrediction, and 110 targets were identified with PharmMapper. We ultimately obtained 205 drug targets after data integration. Similarly, 2401 and 340 targets linked with thrombocytopenia were retrieved from GeneCards and DisGeNET, respectively. Ultimately, we retained 295 disease targets after removing the duplicate targets obtained of these two database analyses. The drug targets and the disease targets intersected to generate 17 potential therapeutic targets for TMEA treatment of thrombocytopenia. These targets are shown in Figure 2A: mTOR, SRC, ABL1, TLR2, PRKCQ, FLI1, MAD2L1, B2M, IVD, E2F1, MS4A1, CA2, PDGFRB, ALOX5, XDH, BCL2L1, and ACHE.

### Topology Analysis on PPI Network and Obtainment of Hub Targets

The 17 therapeutic targets were analysed using the STRING database. Meeting the criterion of a confidence score greater than 0.4, the desired target data were imported into Cytoscape_v3.7.1 to generate an interaction network, which contained 10 nodes and 21 edges. In the network, a node represented a target. The smaller the degree value was, the closer the node colour was to orange. Moreover, the larger the degree value was, the closer the node colour was to blue. Additionally, the edges among the nodes represented PPIs. The thickness of the line denoted the intensity of the PPI, as shown in Figure 2B.

Then, we performed a topology analysis off all nodes in the network using Cytoscape to identify the hub targets. For each node (target), the degree value was set to be greater than 4.5, the betweenness centrality was determined to be greater than 0.08842593, and the closeness centrality was determined to be greater than 0.518092105. With these criteria, 4 hub targets, namely, mTOR, SRC, ABL1, and TLR2, were identified, as shown in Table 2.

**TABLE 2:** Hub targets of TMEA against thrombocytopenia.

**Name**	**Betweenness centrality**	**Closeness centrality**	**Degree**
mTOR	0.22361111	0.81818182	7
SRC	0.09861111	0.75000000	6
ABL1	0.15000000	0.69230769	6
TLR2	0.19074074	0.69230769	5

### Signaling Pathway Prediction Based on GO and KEGG Analyses

To predict the mechanism underlying TMEA action on thrombocytopenia, we conducted GO and KEGG analyses through the DAVID platform. The GO analysis results showed that a total of 75 GO terms were related to thrombocytopenia treatment with TMEA. Of these GO annotations, 54 terms were linked to the BP category and mainly involve protein autophosphorylation, regulation of actin cytoskeleton organization, intrinsic apoptotic signalling pathway in response to DNA damage, positive regulation of fibroblast proliferation, and positive regulation of apoptotic process. Nine terms were related to the CC category and mainly involve the cytosol, extracellular space, perinuclear region of cytoplasm, nuclear membrane, and intrinsic component of plasma membrane. Twelve terms were associated with the MF category and mainly involve protein binding, protein kinase activity, protein kinase binding, protein tyrosine kinase activity, and protein homodimerization activity. In total, 15 BP terms, 5 CC terms, and 5 MF terms showing the greatest enrichment with TMEA-related processes were ranked separately in ascending order of P-values, as shown in Figure 2C. Furthermore, the results of the KEGG analysis revealed that 14 pathways might be involved in TMEA regulatory action in thrombocytopenia, mainly the PI3K-Akt signalling pathway; pathways in glioma, cancer, and chronic myeloid leukaemia; the ErbB signalling pathway; and pathways in prostate cancer and HTLV-I infection. The 10 most-enriched pathways were arranged in ascending order of P-values, as shown in Figure 2D.

### Molecular Docking Assessment

**FIGURE 3:**
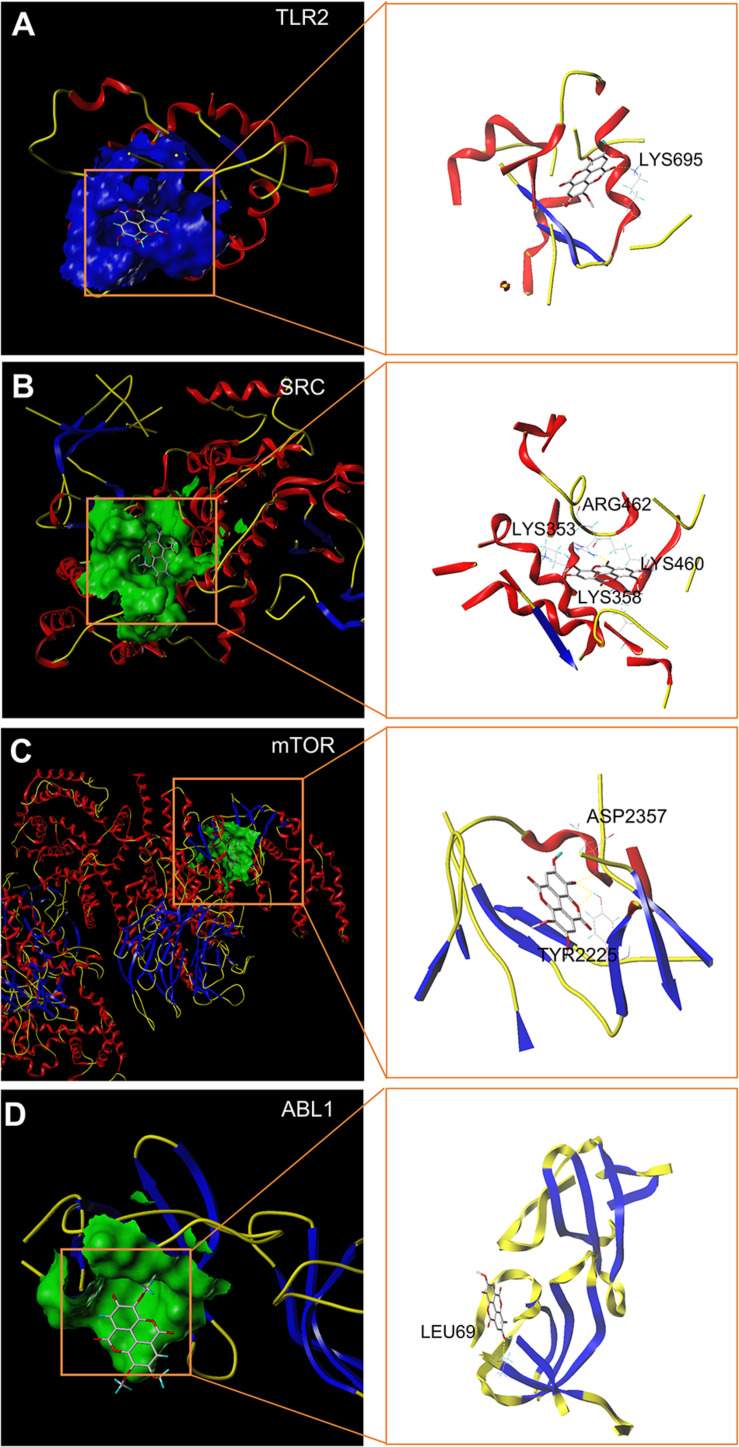
*In silico* molecular docking of TMEA to hub targets. **(A)** TMEA bound to TLR2 protein (1FYW) *via* the active residue LYS695. **(B)** TMEA bound to the SRC protein (1YOJ) *via* active sites ARG462, LYS353, LYS358, and LYS460. **(C)** TMEA bound to mTOR protein (4JT6) *via* the active residues ASP2357 and TYR2225. **(D)** TMEA bound to ABL1 protein (5NP2) at the active site LEU69.

Molecular docking stimulation was used to estimate the binding abilities of TMEA to the 4 hub targets (mTOR, SRC, ABL1, and TLR2). In this study, a docking score (total score) ≥4.52 indicated that the molecule showed binding affinity for the target, and a docking score >5.0 was deemed to indicate high binding activity (Hsin et al., 2013, 2016; Gu et al., 2020). As shown in Figure 3, mTOR and SRC exhibited strong affinities for TMEA, with docking scores of 6.65 and 6.00, respectively.

### Molecular Dynamics Evaluation

**FIGURE 4:**
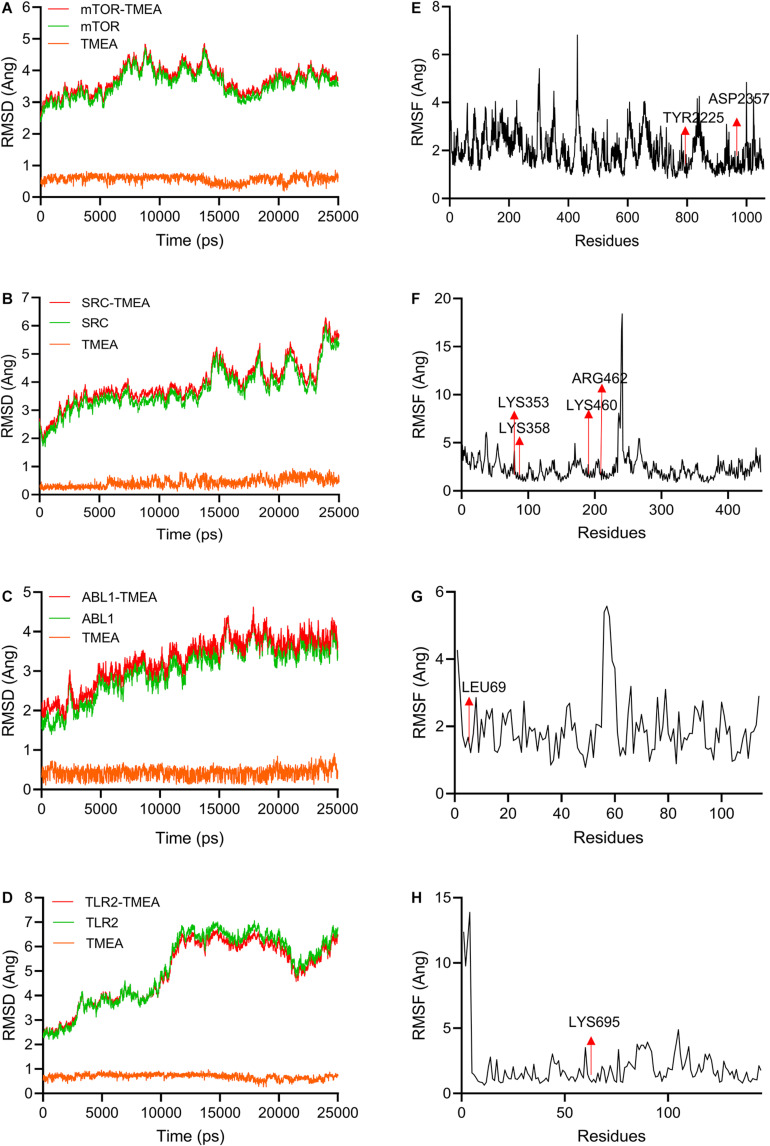
Molecular dynamics simulations (25 ns) used to evaluate TMEA-target interactions. **(A–D)** RMSD values of TMEA binding to mTOR, SRC, ABL1, and TLR2, respectively. **(E–H)** RMSF values of TMEA binding to mTOR, SRC, ABL1, and TLR2, respectively.

To validate these molecular docking stimulation results, we performed 25-ns molecular dynamics simulations and calculated the binding free energies for each complex formation. In this study, the most favourable docking conformations were selected for use in the MD simulations. A 25-ns MD simulation for each system was performed to determine the stable conformation. The RMSDs of mTOR and TMEA fluctuated near ∼4 Å and ∼0.5 Å after 15 ns and 3 ns, respectively (Figure 4A). SRC and TMEA remained stable at ∼5 Å and ∼0.5 Å after 15 ns and 5 ns, respectively (Figure 4B). For the system of ABL1 complexed with TMEA, equilibrium was observed after 20 ns and 1 ns, and the RMSD values were ∼4 Å and ∼0.5 Å (Figure 4C). Similarly, the RMSD values for TLR2 and TMEA fluctuated between ∼7 and 0.8 Å after 10 ns and 5 ns, respectively, which indicated that the complex reached a stable state (Figure 4D). Moreover, for subsequent analyses of residue contribution to each system in the simulation, the root-mean-square fluctuation (RMSF) values versus the residue numbers of all the compounds are shown in Figures 4E–H (Supplementary Videos 1–4). The key residues, including TYR2225, ASP2357, LYS353, LYS358, LYS460, ARG462, LEU69, and LYS695, exhibited rigid behaviours with low RMSF values (approximately 1 A ng), which indicated stable complex properties. These results support the reliability of the MD simulation.

On the basis of the MD simulation, binding free energies were calculated using MM-GBSA methods to assess the energy parameters of the four representative complexes. The binding free energy vales for the complexes are listed in Table 3. The results showed that Δ*G*_bind_ of TMEA binding with mTOR, SRC, ABL1, and TLR2 as determined by MM/GBSA was –35.08, –29.95, –25.09, and –19.02 kcal/mol, respectively.

**TABLE 3:** The binding free energies and the individual energy components (kcal/mol).

**Method**	**Protein**	**ΔG_vdw_**	**ΔG_eel_**	**ΔG_egb_**	**ΔG_esurf_**	**ΔG_bind_**
MM/GBSA	mTOR	–46.8639	–32.7011	49.8815	–5.4013	–35.0848
	SRC	–39.6997	–9.8351	24.1755	–4.5919	–29.9511
	ABL1	–27.3466	–26.5245	32.1618	–3.3804	–25.0897
	TLR2	–29.9988	–11.8640	27.1291	–4.2873	–19.0210

### Megakaryocyte-Like Cells Counting

**FIGURE 5:**
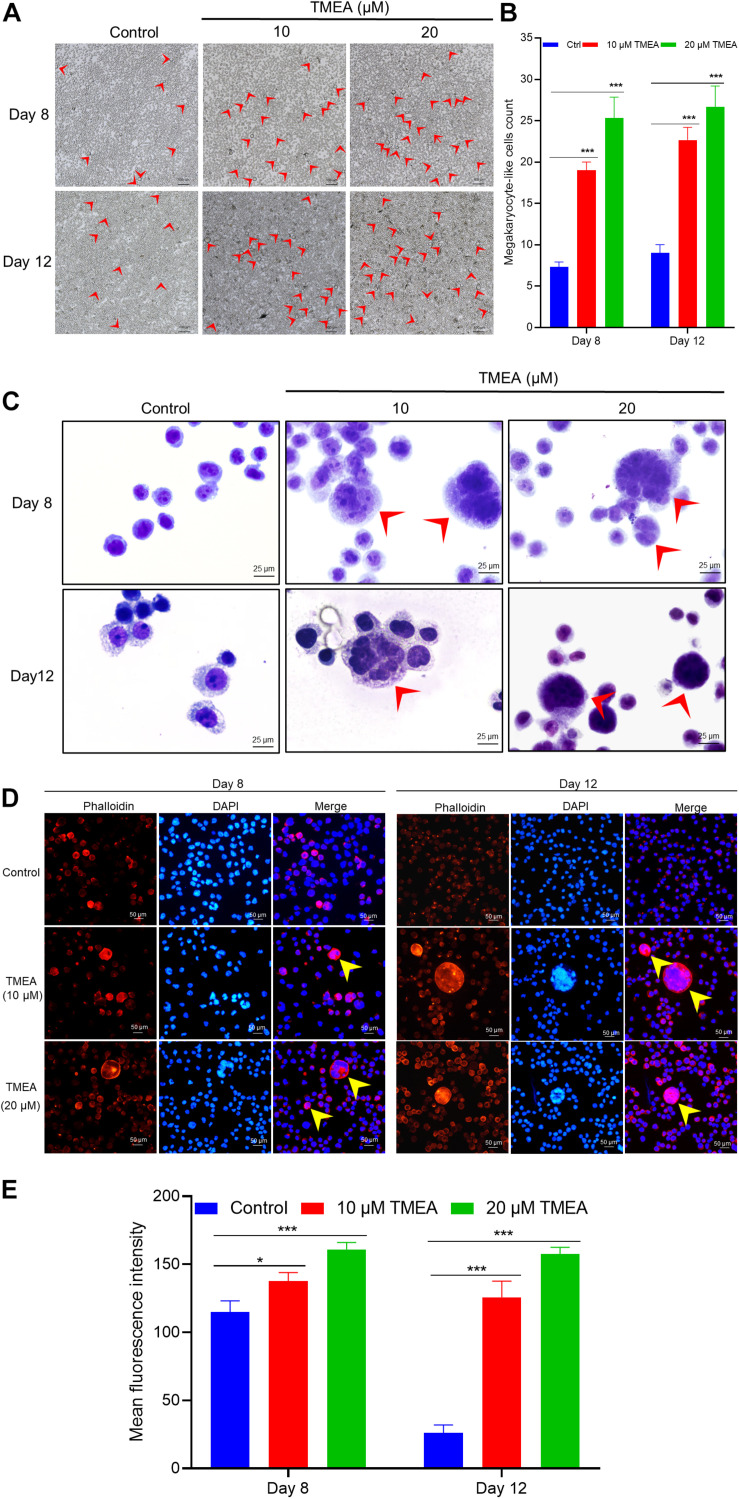
TMEA-induced HEL cell differentiation/polyploidization (megakaryocytic commitment). **(A)** Megakaryocyte-like HEL cell count on the 8th and 12th days of treatment using a microscope at 100× magnification. The megakaryocyte-like HEL cells in the TMEA treated groups were significantly greater than that in the control cells on these two indicated days. **(B)** The histogram displays the statistical result of megakaryocyte-like HEL cell count. *** *P* < 0.001, vs. the control. **(C)** HEL cell nucleotype as determined with Giemsa staining under a microscope at 400× magnification. **(D)** F-actin cytoskeletons of HEL cells are shown with phalloidin staining 8 days and 12 days after treatment with 10 μM or 20 μM TMEA. Arrows designate the salient regions with heavy phalloidin staining. Mean fluorescence intensities in the treated cells at both TMEA concentrations were significantly higher than those in the control cells on the 8th day and 12th day **(E)**. The histograms display the mean fluorescence intensities as determined by statistical analysis. * *P* < 0.05, ** *P* < 0.01, and *** *P* < 0.001, *vs*. the control.

To appraise the overall effect of TMEA on promoting megakaryocyte differentiation, we randomly selected 3 fields viewed under an ordinary microscope to count the megakaryocyte-like cells. As shown in Figure 5A, obvious megakaryocyte-like cells were observed. On the 8th and 12th days, the cells treated with TMEA manifested statistically significant differences (*P* < 0.001) in terms cytomorphology compared to the control cells (Figure 5B).

### Polyploidization

Giemsa staining was used for observing nucleotypes in the HEL cells treated with TMEA. Figure 5C shows apparent nuclear division of the cells treated with TMEA at 10 and 20 μM. This result demonstrates that nuclear division characterizes HEL cell differentiation into megakaryocytes upon TMEA triggering.

**FIGURE 6:**
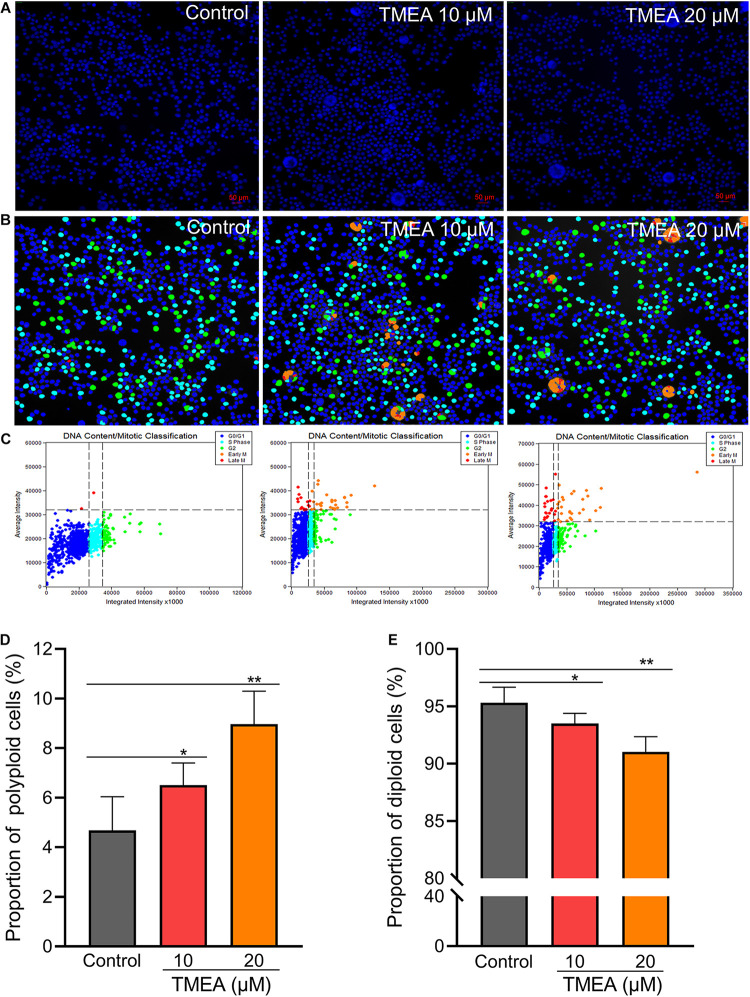
Regulatory effect of TMEA on the cell cycle and DNA ploidy in HEL cells. **(A)** Fluorescence imaging by DAPI staining at 200×. **(B)** and **(C)** Cell cycle classification with DNA ploidy analysis. Blue, aquamarine blue, green, orange, and red cell labels represent the different phases of the cell cycle: G0/G1, S, G2 (≥4 N), early M (≥4 N), and late M phases, respectively. **(D)** The proportion of polyploid cells increased in a concentration-dependent manner 12 days post-TMEA intervention; **(E)** the proportion of diploid cells decreased with increasing TMEA concentration. * *P* < 0.05 and **P < 0.01, *vs*. the control.

HEL cells were treated with 10 or 20 μM TMEA for 12 days. The polyploid proportion in these cells was then detected with a high-content cell imaging analysis system, as shown in Figure 6. Compared with that in the control, the proportion of polyploid cells significantly increased in a concentration-dependent manner after 12 days of TMEA intervention. However, the proportion of diploid cells decreased with increasing TMEA concentration. These results are shown in Figures 6D,E.

### Cytoskeleton Imaging With Phalloidin

**FIGURE 7:**
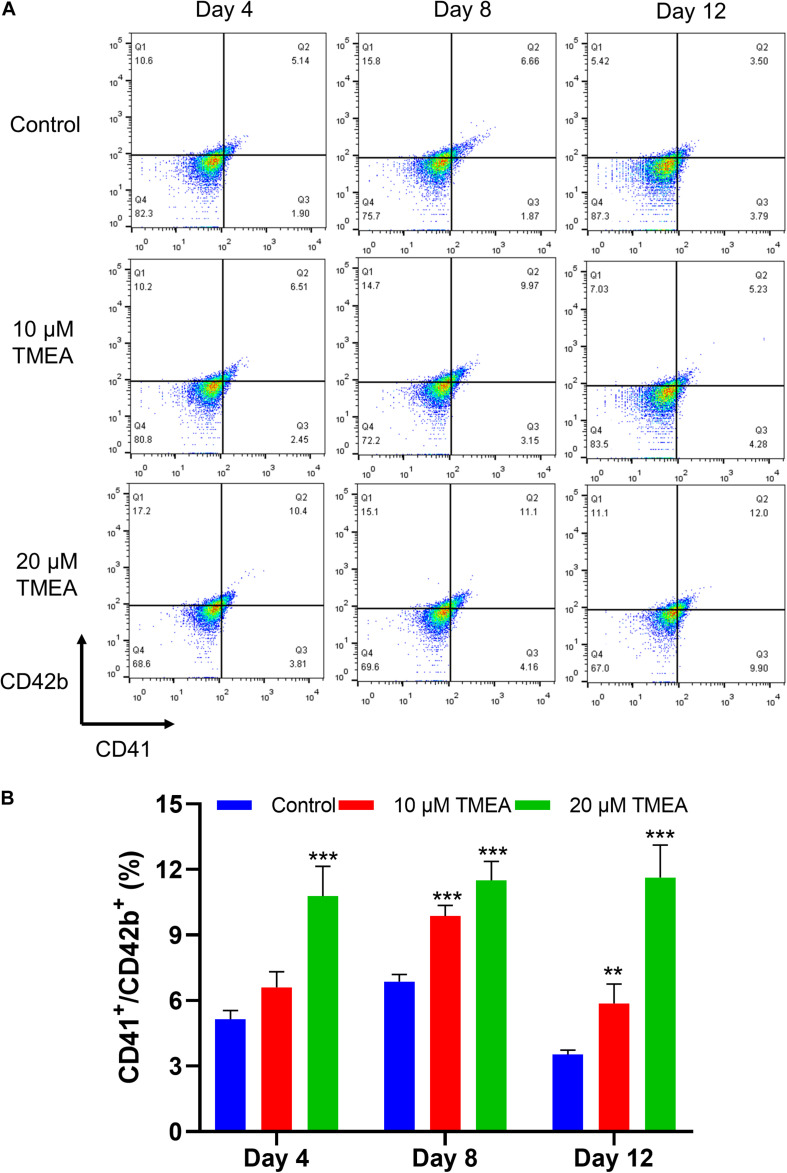
CD41 and CD42b levels in megakaryocytes upon TMEA treatment. **(A)** On the 4th, 8th, and 12th days of TMEA stimulation, the CD41 and CD42b levels were significantly elevated in the treated cells. **(B)** The histograms show the statistical analysis of the cells percent with CD41^+^/CD42b^+^. ** *P* < 0.01, and *** *P* < 0.001, *vs*. the control.

Actin cytoskeleton organization can drive dynamic processes in megakaryocyte maturation, inducing morphological changes such as parapodium and proplatelet formation. In this study, morphological alterations of the HEL cells were evident within 8 to 12 days of TMEA intervention, as determined by filamentous (F-) actin staining and exhibited in Figure 7. Arrows show actin bundles protruding from the cell membrane (Figures 5D,E). These protrusions reflected potential megakaryocyte lineage commitment and proplatelet formation of HEL cells. In contrast, these membrane protrusions were almost absent in the untreated HEL cells (Figure 6).

### Expression of CD41 and CD42b

Changes in the expression of characteristic molecular markers are commonly used to evaluate differentiation and maturation of megakaryocytes (Zhang et al., 2019). The expressions of the cell surface CD41 and the megakaryocytic maturation associated antigen ofCD42b were thus analyzed, and the results showed TMEA obviously improved CD41 and CD42bantibody-stained cell population (Figure 7), confirming the increase ofCD41 and CD42b expressions during the differentiation process of megakaryocytes.

### Reactive Oxygen Species (ROS) Level

**FIGURE 8:**
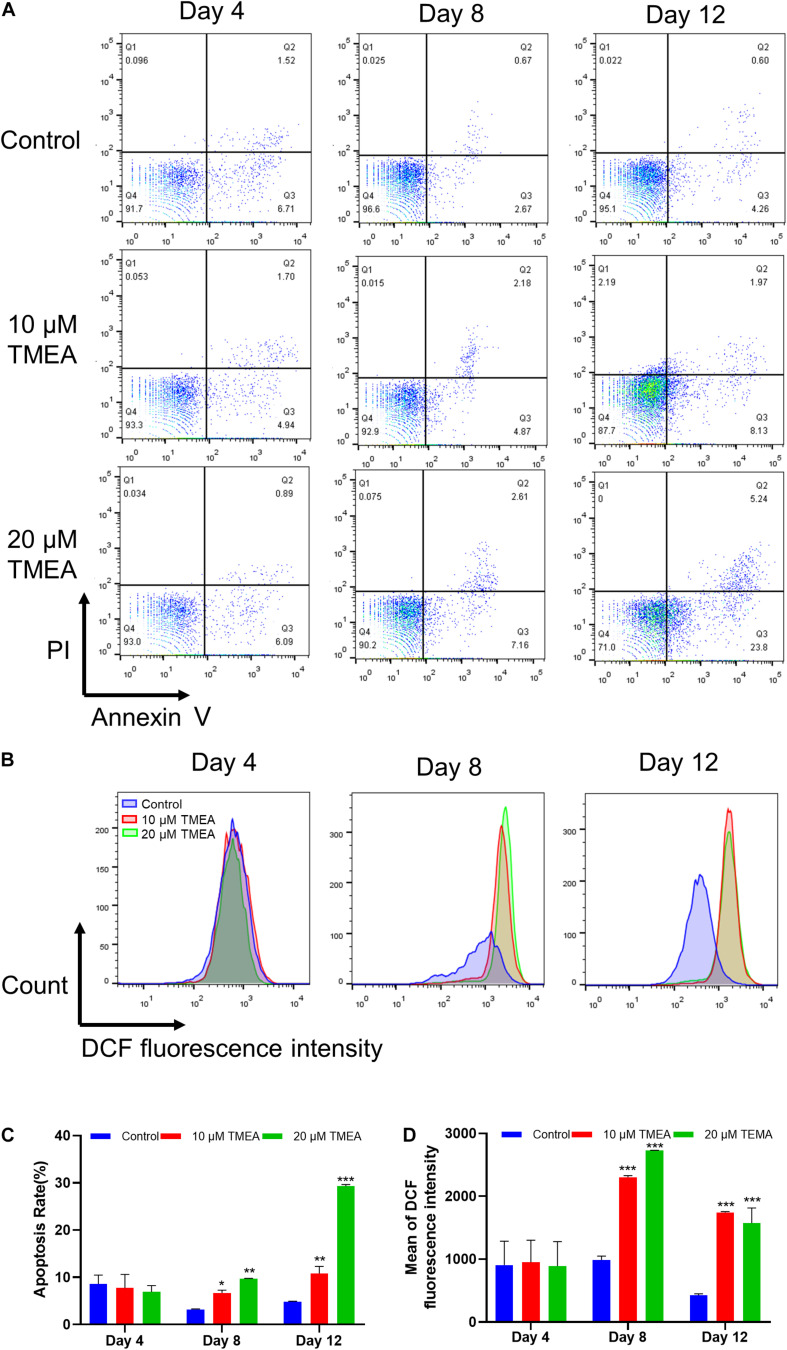
Apoptosis and ROS level by TMEA in HEL cells. **(A)** Apoptotic cells of HEL cells treated TMEA concentrations were measured by flow cytometry using an Annexin V-FITC/PI apoptotic detecting kit. **(B)** Intracellular ROS generation in HEL cells using flow cytometry analysis. **(C)** Quantitative analysis of Annexin V^+^ in different treatments on the 4th, 8th, and 12th days. * *P* < 0.05, ** *P* < 0.01, and *** *P* < 0.001, *vs*. the control. **(D)** Quantitative analysis of ROS generation in different treatments on the 4th, 8th and 12th days. *** *P* < 0.001, *vs*. the control.

ROS generation could play pivotal roles in regulating the differentiation of megakaryocytes (Yang et al., 2019). The results from flow cytometry assay using DCFH-DA probe showed that TMEA induced excessive ROS production in megakaryocytes on the 8th and 12th days (Figure 8A),which was in a concentration-dependent manner (Figure 8C). This result consistently confirmed oxidative stress could be induced by TMEA in megakaryocytes, thus resulting in the accelerated thrombopoiesis.

### Apoptosis Analysis

Proplatelet formation requires localized apoptosis to facilitate the cytoskeletal rearrangements required for platelet shedding (De et al., 2002). The results from flow cytometry assay using Annexin V/PI staining showed that TMEA induced excessive apoptosis in megakaryocytes on the 8th and 12th days (Figure 8B), which was in a concentration-dependent manner (Figure 8D).

### Transcription Factor Analysis Using Immunofluorescence

**FIGURE 9:**
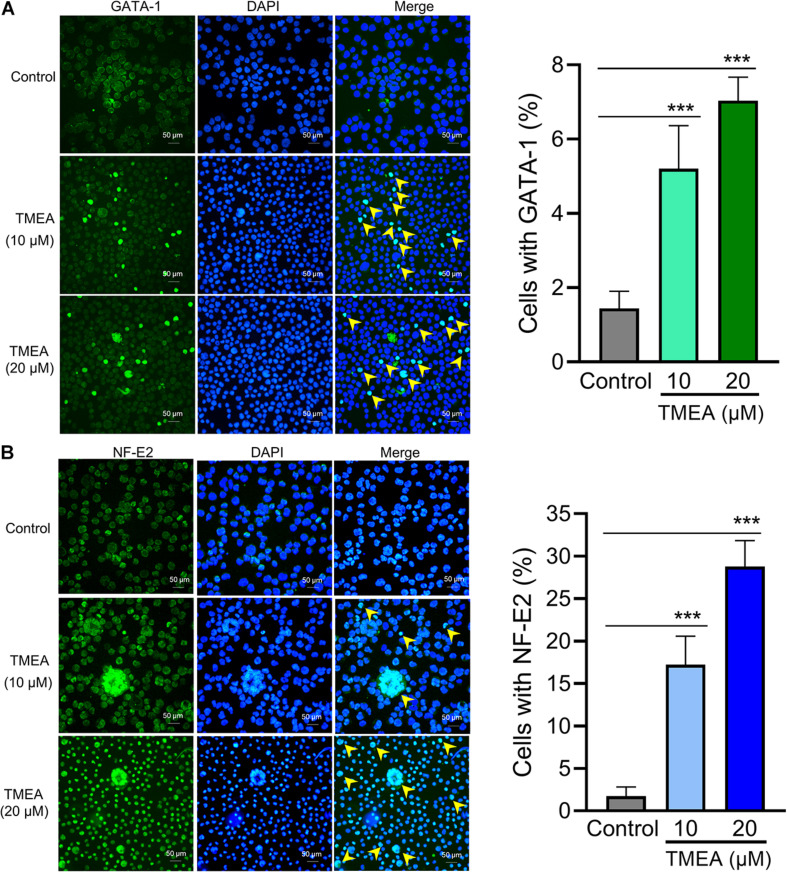
Transcription factor regulation by TMEA in HEL cells. GATA-1 **(A)** and NF-E2 **(B)** expression in HEL cells is shown by fluorescence intensity. After stimulation with 10 μM or 20 μM TMEA, GATA-1, and NF-E2 expression was significantly increased in the treated cells on the 12th day compared with the control. Arrows show the distinct regions with heavy anti-GATA-1 and anti-NF-E2 staining separately. The histograms show the statistical analysis of cells with GATA-1 or NF-E2 fluorescence signal. * *P* < 0.05, ** *P* < 0.01, and *** *P* < 0.001, *vs*. the control.

The transcription factors GATA-1 and NF-E2 play essential roles in megakaryocytic differentiation and maturation as well as proplatelet formation, associating upstream molecular signalling with downstream transcript expression. After TMEA treatment at 10 μM or 20 μM, GATA-1 and NF-E2 expression, quantified by fluorescence intensity, was significantly increased in the treated HEL cells on the 12th day compared with that in the control cells. The arrows show the protein expression of GATA-1 and NF-E2 (Figures 9A,B). This result warrants to subsequent investigation on the molecular mechanism of TMEA in promoting HEL cell (megakaryocyte) differentiation.

### Pathway Activity Detected by Western Blot

**FIGURE 10:**
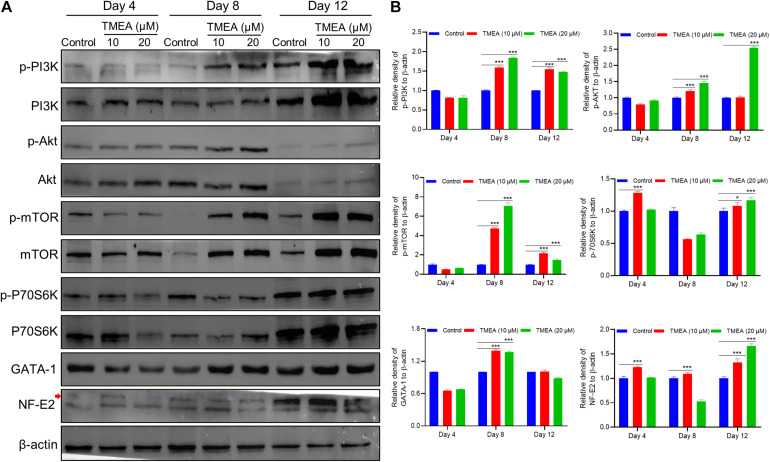
TMEA-triggered activation of the signalling cascade in HEL cell differentiation. **(A)** The hub protein expression in the theoretical pathway was validated by western blot analysis and denoted by relative density. On the 4th, 8th, and 12th days of TMEA stimulation, the phosphorylation levels of PI3K, AKT, mTOR, and P70S6K were significantly elevated in the treated cells. Additionally, the expression of transcription factors GATA-1 and NF-E2 was markedly increased in the treated cells compared with the control cells. **(B)** The histograms show the statistical analysis of the relative densities. * *P* < 0.05 and *** *P* < 0.001, *vs*. the control.

Since the *in silico* prediction results emphasized the PI3K/Akt signalling pathway involvement in a latent regulatory mechanism of TMEA action, we measured the expression of the hub proteins in this pathway, which are diverse and embody TMEA-prompted pathway regulation: (p-)PI3K, (p-)Akt, (p-)mTOR, (p-)P70S6K, GATA-1, and NF-E2 (Figure 10). On the 4th day, phosphorylation levels of PI3K, AKT, and mTOR were no significant changes in the TMEA treated cells. On the 8th and 12th days of TMEA stimulation, phosphorylation levels of PI3K, AKT, and mTOR, P70S6K markedly increased in the treated cells, showing the same trend as transcription factor expression (*P* < 0.05 or 0.001). These results indicate that TMEA stimulation can promote megakaryocyte differentiation/maturation by activating the PI3K/AKT/mTOR/P70S6K/GATA-1/NF-E2 cascade.

### *In vivo* Blood Platelet Level

**FIGURE 11:**
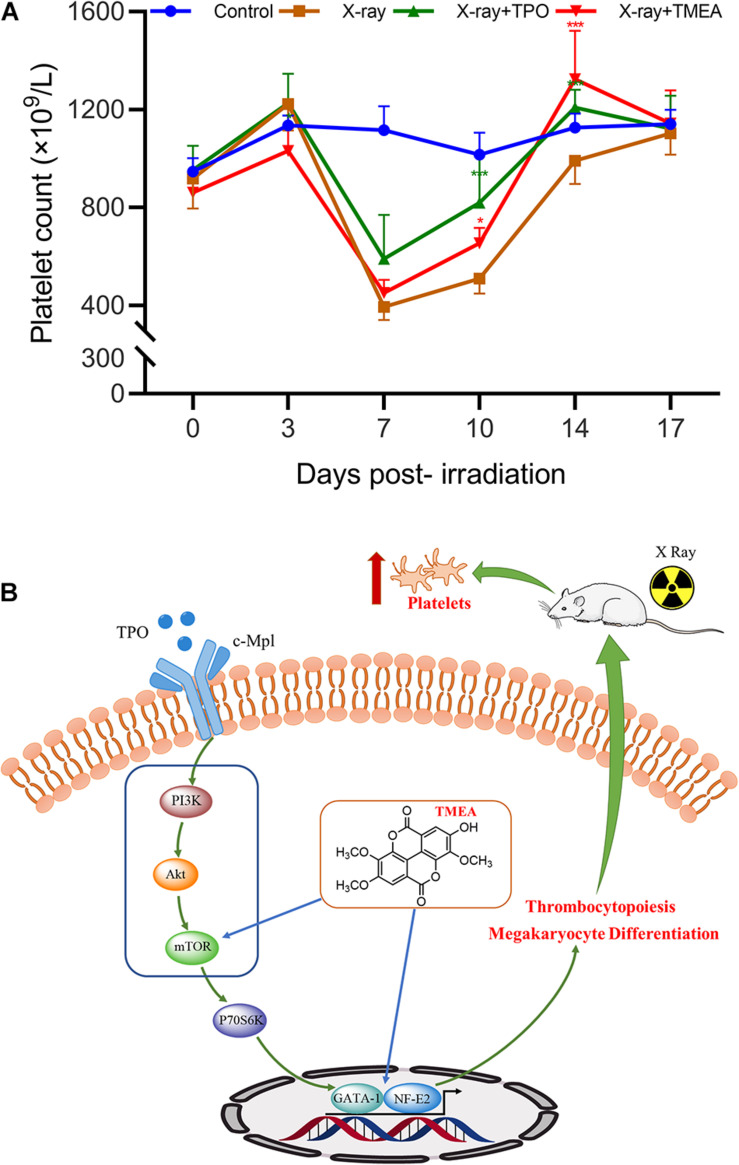
*In vivo* platelet recovery by TMEA administration and its underlying molecular mechanism. **(A)** The blood platelet count (PLT) in the treated mice reflected the therapeutic effect of TMEA on X-ray-induced thrombocytopenia. Compared to that in the control, the PLT in the model mice was reduced to the minimum on the 7th day post-irradiation. From the 7th to 14th days, the PLT in the TMEA-injected mice was higher than that in the model mice. The line chart exhibits the statistical analysis of PLT. * *P* < 0.05, ** *P* < 0.01, and *** *P* < 0.001, *vs*. the X-ray group. **(B)** The PI3K/Akt/mTOR/P70S6K/GATA-1/NF-E2 pathway may be part of the key molecular mechanism of TMEA action on megakaryocytic differentiation and platelet production.

To confirm the *in vivo* platelet-increasing effect of TMEA, we injected X-ray-exposed mice intraperitoneally with TMEA. Owing to the X-ray exposure, the blood platelet count in the model mice declined to a minimum on the 7th day after irradiation, while that of the control mice was unchanged. TMEA administration notably inhibited X-ray-induced blood platelet reduction (*i.e.*, thrombocytopenia) and boosted platelet recovery from days 7 to 14 (*P* < 0.001), as shown in Figure 11A.

## Discussion

Given the physiological function of platelets in blood coagulation, haemorrhage is the primary concomitant symptom of thrombocytopenia. Although routine clinical remedies in response to thrombocytopenic bleeding are capable of boosting platelet recovery and inhibiting haemorrhagic progression, serious side effects, high medical expenses and high recurrence rates caused by these therapies may place large burdens on the patient population (Delaney et al., 2016). In this regard, phytoproduct intervention as a highlighted alternative may create opportunities for achieving better outcomes of thrombocytopenia treatment (Gao and Chong, 2012; Sun et al., 2018).

Traditional Chinese herbs comprise a treasure trove of natural medicinal products. TMEA, an SO polyphenol, was deduced to be a core compound that might govern the thrombopoiesis-augmenting mechanism of a TCM since the chemical traits of this compound meet the aforementioned criteria. To probe this assumption, in this study, we employed *in silico* analysis (combining a network pharmacology approach and molecular docking simulation) followed by experimental investigations (*in vivo and in vitro*).

Useful for the multitargeted regulatory patterns of natural products, the network pharmacology method enabled the integration multiple of multiple factors for determining a TMEA-mediated signalling map, similar to that in other reported studies (Lv et al., 2014; Zhu et al., 2018; Tu et al., 2020; Wang Z. Y. et al., 2020; Wang W. et al., 2020). Using target prediction, we identified 17 latent target proteins in the compound-target-disease interrelated network: mTOR, SRC, ABL1, TLR2, PRKCQ, FLI1, MAD2L1, B2M, IVD, E2F1, MS4A1, CA2, PDGFRB, ALOX5, XDH, BCL2L1, and ACHE. Among these targets, 4 diverse hub targets, mTOR, SRC, ABL1, and TLR2, were identified in the PPI network, which was constructed with 10 nodes and 21 edges. Moreover, the *in silico* docking data of TMEA with the hub targets were used in modelling simulations to estimate binding affinities, showing that the docking score of mTOR with TMEA was 6.65, the highest appetency among the tested compounds. These findings laid the foundation for signalling pathway inference and follow-up experimental validation. We conjectured that the molecular mechanism of TMEA action in thrombocytopenia is likely related to the TMEA interaction with the hub protein mTOR.

mTOR, mechanistic target of rapamycin, is a serine/threonine protein kinase that regulates various cellular functions, including cell survival, growth, proliferation, differentiation, apoptosis, and homeostasis maintenance (Laplante and Sabatini, 2012). Especially during multilineage haematopoiesis, mTOR exhibits distinct functions, even playing opposite roles in different phases. For example, mTOR activation was indispensable for the maintenance of haematopoietic stem cells (HSCs) but not haematopoietic progenitors (Zhou et al., 2016). In terms of erythroid cell commitment, mTOR inhibition by rapamycin significantly improved erythroid cell maturation and anaemia in a β-thalassemia mouse model (Zhang et al., 2014), whereas suppression of mTOR signalling markedly reduced the proportion of erythroid progenitor cells in a population of wild-type bone marrow cells (Liu et al., 2020). During megakaryocytic development, mTOR overexpression might inhibit megakaryocyte differentiation by triggering abnormal autophagy, as indicated when the autophagy-related gene ATG7 was knocked out (Drayer et al., 2006; Liu et al., 2011; Wang et al., 2016; Malik et al., 2018; Sun and Shan, 2019). Additionally, mTOR blockade with rapamycin suppressed the maturation of both adult and neonatal megakaryocytes; the former showed inhibited polyploidization and cytoplasmic maturation, and the latter showed no ploidy changes (Hou et al., 2018). These outcomes emphasize the significance of mTOR biofunction in haematopoiesis, notably megakaryocyte maturation and platelet production related to our study. Therefore, it is easy to understand that mTOR modulation by TMEA may be a pivotal point in TMEA-controlled signal transduction.

In addition to identifying the targets of TMEA action, we obtained 14 potential pathways using KEGG pathway enrichment analysis. Of these pathways, the PI3K/Akt signalling pathway caught our attention not only because of its top rank among the enriched pathways but also because of its linkage to mTOR as a downstream signalling molecule. PI3K/Akt is an essential pathway mediating cell survival, differentiation, proliferation, apoptosis and migration (Hers et al., 2011). Disorders of this pathway can be found in various human diseases, including cancer, diabetes, cardiovascular diseases, nervous system diseases, and haematopoietic diseases (Buitenhuis and Coffer, 2009; Polak and Buitenhuis, 2012). For platelet formation, the intracellular PI3K/Akt pathway can be activated by multiple haematopoietic factors, such as stem cell factor, platelet-derived growth factor, interleukins, and thrombopoietin (TPO). These extracellular regulators combine with their membrane receptors to maintain homeostasis and promote committed differentiation of HSCs and secondary progenitors, leading to megakaryopoiesis and thrombopoiesis (Nakao et al., 2008; Vainchenker and Raslova, 2020). As intracellular signal transducers, PI3K and Akt together constitute a signalling cascade transmitting a stimulative signal from the activated receptor to subsequent signalling molecules. mTOR, one of the downstream signalling molecules of Akt, is activated by its interaction with Akt, which phosphorylates P70S6K and further genetically upregulates the expression of transcription factors, including GATA-1 and NF-E2. Expression of GATA-1, an important transcription activator in megakaryocytes, is conducive to NF-E2 expression and governs the transcriptional activity of FOG-1 through interaction. These regulatory processes positively affect megakaryocyte maturation and proplatelet formation by amplifying F-actin expression (important in cytoskeleton organization/remodelling) and assembly in a PDK1-dependent manner (Bury et al., 2016; Johnson et al., 2016; Geue et al., 2019). Accordingly, it is essential and feasible to augment megakaryopoiesis/thrombopoiesis cells to enhance the PI3K/Akt/mTOR/P70S6K/GATA-1/NF-E2 signalling pathway. Moreover, from the pathological perspective, thrombocytopenia development correlates with abnormalities in megakaryocyte production, growth, and differentiation (Flaumenhaft et al., 2009; Wang et al., 2019). These cytological variations result from cell signalling dysfunction, particularly affecting the PI3K/Akt/mTOR pathway and its downstream cascades (Raslova et al., 2003). We can thereby surmise that this pathway may play a critical role in thrombocytopenia treatment with TMEA, acting in a core molecular mechanism. In addition, the GO analysis revealed BPs such as protein autophosphorylation, regulation of actin cytoskeleton organization, and platelet-derived growth factor receptor-beta signalling pathway (Figure 2), which also affect megakaryopoiesis/thrombopoiesis-associated signalling pathways, supporting the stated importance of the PI3K/Akt/mTOR pathway.

The evidence from *in vitro* and *in vivo* experiments corroborates the predicted results. Because platelets originate from the differentiation of megakaryocytes, polyploidy occurs, and multilobulated nuclei appear when megakaryocytes mature (Cullmann et al., 2021). These phenotypes were observed by differentiated cell counting, Giemsa staining and DAPI staining after TMEA intervention. Furthermore, cytoskeletal staining with phalloidin also showed the ability of TMEA to promote megakaryocyte commitment to proplatelets in our study on the basis of the close association between actin and cytoplasmic division (Rojnuckarin and Kaushansky, 2001). Cell shape maintenance and change are dependent on 2 cytoskeleton systems, the microtubule cytoskeleton and F-actin cytoskeleton. In mature megakaryocytes, the microtubule cytoskeleton propels proplatelet formation through microtubule polymerization or reorganization, while the F-actin cytoskeleton is critical for stabilizing cell shape at a resting state, organizing the topology of surface receptors, and driving cytoplasm fragmentation as well as platelet spreading. Hence, F-actin expression can be used to quantitatively estimate megakaryocyte differentiation/maturation. Previous ultrastructural analysis of proplatelet-committed megakaryocytes by phalloidin staining helped reveal the dispersion of actin filaments, showing a similar tendency to our data (Rojnuckarin and Kaushansky, 2001). All platelets are the progeny of megakaryocytes, and megakaryocytes that produce functional platelets are marked as CD41^+^CD42b^+^(Schulze and Italiano, 2016; Zhang et al., 2019). In this study, the count of CD41^+^CD42b^+^cells were upregulated during megakaryocyte differentiation/maturation for the 12 consecutive days.

The following investigations were based on the *in vitro* activity verification above. We further tested the therapeutic effect of TMEA on irradiation-induced thrombocytopenia in mice. In this *in vivo* study, TMEA injection resulted in a significant increase in peripheral platelet count, especially 7 and 10 days after X-ray irradiation of the mice. On account of bone marrow haematopoietic dysfunction after whole-body irradiation, the results reveal TMEA action on thrombopoiesis and may partly reflect its linkage to megakaryocyte differentiation/maturation. Because TMEA treatment for thrombocytopenia was experimentally verified to be effective, we explored the key regulatory mechanism of TMEA in thrombopoiesis. As predicted *in silico*, the adjustment to the PI3K/Akt/mTOR/P70S6K/GATA-1/NF-E2 signalling pathway may be instrumental in facilitating thrombopoiesis by TMEA (Figure 11B). Therefore, we detected critical protein phosphorylation to decipher whether TMEA functioned through this proposed pathway. The results suggest that phosphorylated proteins such as p-PI3K, p-Akt, p-mTOR, and p-P70S6K were significantly upregulated in TMEA-treated HEL cells compared to those in the control cells. Moreover, TMEA intervention enhanced the expression of the transcription factors NF-E2 and GATA-1 in HEL cells, which was confirmed by both western blot analysis and immunofluorescence staining. These findings not only demonstrate a bridge created between TMEA activity and the predicted mechanism but also demonstrate the indispensable functions of NF-E2 and GATA-1 as transcriptional regulators in megakaryocyte development (Takayama et al., 2010; Rost et al., 2018).

Moreover, the ROS generation could play pivotal roles in regulating the megakaryocyte differentiation (Chen et al., 2013; Yang et al., 2019). In the present study, the elevation in cellular ROS level due to TMEA treatment, confirming the differentiation and maturation of TMEA-induced megakaryocytes were accompanied by the increase of ROS. Also, apoptosis is important for megakaryocyte development and thrombopoiesis. Some scholars believed that it was necessary to inhibit the apoptosis of megakaryocytes in order to survive and proceed safely through the process of platelet shedding (Josefsson et al., 2011), while some others believed that megakaryocytes could shed platelets by promoting apoptosis (De et al., 2002). A preliminary study found no obvious apoptosis on the 4th day, whereas on the 8th and 12th days, the cells treated with TMEA showed concentration-dependent apoptosis. We suspected that TMEA had a protective effect on megakaryocytes in the early stage of TMEA intervention. Over time, megakaryocytes shed proplatelets by increasing apoptosis.

Taken together, our investigations profile the characteristics of TMEA action on megakaryocyte maturation/platelet production, from *in vitro* to *in vivo* experiments and from virtual prediction to experimental validation. However, in-depth explorations still need to be carried out, including pharmacokinetic analysis of TMEA administration and human primary megakaryocytes studies of TMEA therapy for thrombocytopenia.

## Conclusion

In conclusion, the present study for the first time predicted the targets and signalling cascades of TMEA action against thrombocytopenia and determined conclusively that the regulation of the PI3K/Akt/mTOR/P70S6K/GATA-1/NF-E2 signalling pathway lies at a nexus of TMEA targeting and megakaryocytic differentiation into proplatelets. Our findings provide novel insight into potential thrombopoiesis-stimulating medications.

## Data Availability Statement

The raw data supporting the conclusions of this article will be made available by the authors, without undue reservation.

## Ethics Statement

The animal study was reviewed and approved by the Ethics Committee of Southwest Medical University.

## Author Contributions

HL and XS performed the network pharmacological analysis. HL and XJ wrote the manuscript. HL, LW, and FH performed the *in vivo* experiments. YS, XJ, HL, and JL performed the *in vitro* experiments. XJ, NJ, and HL performed the statistical analysis. YL, AW, and DQ provided useful suggestions on the methodology. JZ and LY carried out UPLC-MS analysis. JW, QM, and JY designed the study, revised the manuscript, and approved the final proof as corresponding authors. All authors read and approved the final manuscript.

## Conflict of Interest

The authors declare that the research was conducted in the absence of any commercial or financial relationships that could be construed as a potential conflict of interest.

## Publisher’s Note

All claims expressed in this article are solely those of the authors and do not necessarily represent those of their affiliated organizations, or those of the publisher, the editors and the reviewers. Any product that may be evaluated in this article, or claim that may be made by its manufacturer, is not guaranteed or endorsed by the publisher.

## Footnotes

1
http://www.swissadme.ch

2
https://pubchem.ncbi.nlm.nih.gov/

3
http://swisstargetprediction.ch/

4
http://www.lilab-ecust.cn/pharmmapper/

5
https://www.genecards.org/

6
http://www.disgenet.org/

7
http://string-db.org

8
http://www.omicshare.com/

9
https://www.rcsb.org/
